# *Escherichia coli* producing AmpC DHA-1 bacteraemia in neutropenic leukemic patient: continuous infusion ceftazidime/avibactam as a carbapenem sparing regimen

**DOI:** 10.1093/jacamr/dlaf109

**Published:** 2025-06-18

**Authors:** S Giuliano, A Piccirilli, J Angelini, F Patriarca, R Fanin, L Martini, T Semenzin, M Fanin, S Lanini, R A Bonomo, M Perilli, C Tascini

**Affiliations:** Infectious Diseases Clinic, Azienda Sanitaria Universitaria del Friuli Centrale (ASUFC), Udine, Italy; Department of Biotechnological and Applied Clinical Sciences, University of L’Aquila, L’Aquila, Italy; Department of Medicine (DMED), University of Udine, Udine, Italy; Clinical Pharmacology and Toxicology Institute, University Hospital Friuli Centrale ASUFC, Udine 33100, Italy; Department of Medicine (DMED), University of Udine, Udine, Italy; Haematological Clinic and Transplant Centre, Azienda Sanitaria Universitaria del Friuli Centrale (ASUFC), Udine, Italy; Department of Medicine (DMED), University of Udine, Udine, Italy; Haematological Clinic and Transplant Centre, Azienda Sanitaria Universitaria del Friuli Centrale (ASUFC), Udine, Italy; Infectious Diseases Clinic, Azienda Sanitaria Universitaria del Friuli Centrale (ASUFC), Udine, Italy; Department of Medicine (DMED), University of Udine, Udine, Italy; Infectious Diseases Clinic, Azienda Sanitaria Universitaria del Friuli Centrale (ASUFC), Udine, Italy; Department of Medicine (DMED), University of Udine, Udine, Italy; Haematological Clinic and Transplant Centre, Azienda Sanitaria Universitaria del Friuli Centrale (ASUFC), Udine, Italy; Infectious Diseases Clinic, Azienda Sanitaria Universitaria del Friuli Centrale (ASUFC), Udine, Italy; Department of Medicine (DMED), University of Udine, Udine, Italy; Medical Service and Center for Antimicrobial Resistance and Epidemiology, Louis Stokes Cleveland Veterans Affairs Medical Center, University Hospitals Cleveland Medical Center and Departments of Medicine, Pharmacology, Molecular Biology, and Microbiology, Case Western Reserve University, Cleveland, OH, USA; Department of Biotechnological and Applied Clinical Sciences, University of L’Aquila, L’Aquila, Italy; Infectious Diseases Clinic, Azienda Sanitaria Universitaria del Friuli Centrale (ASUFC), Udine, Italy; Department of Medicine (DMED), University of Udine, Udine, Italy

## Abstract

**Background:**

*Escherichia coli* resistant to third-generation cephalosporins, primarily due to the production of ESBLs and AmpC β-lactamases, poses a significant therapeutic challenge, particularly in immunocompromised patients. Ceftazidime/avibactam (CZA) has emerged as a potential carbapenem-sparing option, though data on its efficacy against AmpC-producing Enterobacterales remain limited.

**Methods:**

We report a case of bloodstream infection (BSI) caused by an *E. coli* strain harbouring the plasmid-mediated AmpC enzyme DHA-1 in a neutropenic patient following allogeneic haematopoietic stem cell transplantation. The strain was characterized via whole-genome sequencing and conjugation assays. Therapeutic drug monitoring (TDM) was used to guide a continuous infusion CZA regimen in the context of augmented renal clearance (ARC).

**Results:**

The patient responded favourably to CZA therapy (2.5 g every 8 h via continuous infusion for 9 days), with rapid microbiological clearance and clinical improvement. TDM confirmed therapeutic plasma concentrations of both ceftazidime (29.57 mg/L) and avibactam (5.52 mg/L). Genomic analysis revealed multiple resistance genes (*blaDHA-1*, *qnrB4*, *mphA*, *dfrA7*) and virulence factors, with the isolate identified as *E. coli* ST442, serotype O174:H9. The early switch from meropenem to CZA may have contributed to microbiota preservation and prevented subsequent infection by carbapenemase-producing *Klebsiella pneumoniae*, for which the patient was colonized.

**Conclusions:**

This case illustrates the clinical utility of a carbapenem-sparing strategy guided by TDM in a high-risk, ARC patient with an AmpC-producing *E. coli* BSI. Continuous infusion CZA achieved pharmacokinetic/pharmacodynamic targets associated with therapeutic success, offering a promising alternative to carbapenems while mitigating the risk of resistance development and microbiota disruption.

## Introduction

*Escherichia coli* is one of the six highly virulent and antibiotic resistant bacterial pathogens whose acronym is ESKAPE. The EARS-Net for 2023 reports that more than half of the *E. coli* isolate were resistant to almost one class of antibiotic under surveillance.^1^ Third-generation cephalosporin resistant *E. coli* seems to be a major cause of bloodstream infections (BSIs) in Europe with an estimate of deaths of the total number of resistant strains equal to 20.9%.^2^ Generally, in Enterobacterales, resistance to third-generation cephalosporins is predominantly linked to the production of ESBLs and AmpC-type β-lactamases.^2^ Notably, constitutive overexpression of AmpC β-lactamases in Gram-negative organisms occurs either by deregulation of the *AmpC* chromosomal gene or by acquisition of a transferable *AmpC* gene on a plasmid or other transferable element.^3^ Third-generation cephalosporin resistance in *E. coli* is primarily attributed to the production of ESBLs that spread, more often, by mobile genetic elements or by epidemic plasmids.^4^ The prevalence of ESBLs compared to AmpC in Enterobacterales associated with invasive infections is on the rise, with current estimates indicating a ratio of approximately 10:1.^3^

In terms of treatment, infections caused by Enterobacterales with AmpC production often exhibit poor response to piperacillin–tazobactam,^5,6^ as AmpC is largely unaffected by tazobactam.^7^ Consequently, carbapenems are typically recommended as the first-line treatment for severe infections. However, for less severe infections with a cefepime MIC ≤ 2 mg/L, cefepime could be considered as an alternative.^8^ Ceftolozane–tazobactam has emerged as a promising option to carbapenems, particularly in severe pneumonia cases caused by *Pseudomonas aeruginosa* and ESBL and AmpC-producing Enterobacterales.^9^ There is evidence supporting its efficacy, especially in neutropenic patients. However, there remains a lack of substantial evidence regarding the effectiveness of ceftolozane–tazobactam against AmpC-producing Enterobacterales. In fact, current IDSA guidance does not recommend this drug for treating infections caused by AmpC-producing Enterobacterales.^9^ Ceftazidime/avibactam (CZA), a combination of a well-established third-generation cephalosporin and a novel diazabicyclooctane β-lactamase inhibitor with minimal direct antibacterial effect, significantly enhances the activity of ceftazidime against multidrug-resistant Gram-negative pathogens, including KPC-producing Enterobacterales and certain *P. aeruginosa* strains, thus offering a valuable alternative to carbapenems for both empiric use in high-risk patients and targeted therapy guided by susceptibility profiles.^10^ Neutropenic patients, particularly those undergoing chemotherapy for acute myeloid leukaemia or allogeneic bone marrow transplantation, face substantial mortality risks from BSIs caused by Gram-negative bacteria, including *Pseudomonas* species and Enterobacterales.^11^ Historically, the standard of care for these patients has involved the use of carbapenems. However, prolonged carbapenem use raises concerns regarding the selection of carbapenemase-producing Enterobacterales (CPE) and can contribute to severe dysbiosis, a condition recently associated with acute graft-versus-host disease (aGVHD).^12^

Herein, we present a clinical case of a neutropenic patient who developed a BSI caused by *E. coli* producing AmpC DHA1. The patient exhibited remarkable recovery following treatment with a carbapenem-sparing regimen, based on continuous infusion of ceftazidime/avibactam, and supported by therapeutic drug monitoring (TDM) of this drug, which we routinely perform in our hospital,^13^ and which consists of direct measurement of both ceftazidime and avibactam concentrations in patient’s plasma throughout the antimicrobial treatment.

## Methods

The *E. coli* (UD-2024) strain, isolated from blood culture of a neutropenic patient, showed resistance to ceftazidime, ceftriaxone, amoxicillin–clavulanate, piperacillin–tazobactam and ceftolozane–tazobactam (Table 1). The *E. coli* strain was characterized by whole-genome sequencing using the Illumina MiSeq platform (Illumina Inc., San Diego, USA). Short-read sequencing libraries were prepared with the Illumina DNA Prep Kit following a 2 × 300 bp paired-end protocol. Raw data from sequencing were quality checked using DRAGEN FastQC + MultiQC v3.9.5 (https://basespace.illumina.com/apps/12821810/DRAGEN-FastQC-MultiQC, access date: 18 November 2023) and were subsequently assembled with SPAdes Genome Assembler v3.9.0 (https://basespace.illumina.com/apps/3047044/SPAdes-Genome-Assembler?preferredversion, access date: 18 November 2023). MLST was performed by analysing seven housekeeping genes (*adk*, *fumC*, *gyrB*, *icd*, *mdh*, *purA* and *recA*) using a BLAST-based approach. The ST was assigned according to the Achtman scheme (https://pubmlst.org/bigsdb?db=pubmlst_mlst_sqdef&page=schemeInfo&scheme_id=4).

**Table 1.** Antibiogram of *Escherichia coli* isolated from blood samples

Antibiotic	MIC (mg/L)
Amikacin	S ≤ 4
Amoxicillin/clavulanate	R > 64
Ceftazidime	R > 64
Ceftazidime/avibactam	S ≤ 1
Ceftriaxone	R > 4
Ceftriaxone meningitis	R > 4
Ciprofloxacin	S 0.25
Ertapenem	S ≤ 0.5
Fosfomycin c/G6P	S ≤ 16
Gentamicin	S ≤ 1
Meropenem	S ≤ 0.125
Meropenem meningitis	S ≤ 0.125
Piperacillin/tazobactam	R > 128
Tigecycline	S ≤ 0.25
Trimethoprim/sulfamethoxazole	I = 4
Ceftolozane/tazobactam	R > 8

To investigate the presence of the *bla_DHA-1_* gene within IncFIB/IncFII plasmids, conjugation experiments were performed using *E. coli* J53 as recipient and *E. coli* ST442 as donor. Transconjugants were selected on LB agar plates supplemented with 300 mg/L rifampicin and 100 mg/L cephamycin. The detection sensitivity of the assay was set at ≥5 × 10⁻⁷ transconjugants per recipient. Conjugational transfer of *bla_DHA-1_* was confirmed by PCR using specific primers.

### Creatinine clearance and augmented renal clearance definition

Augmented renal clearance (ARC) was defined according to the criteria proposed by Aaron M. Cook, which identify ARC as a mean creatinine clearance ranging from 170 mL/min to over 300 mL/min.^14^ Creatinine clearance was calculated using the Cockcroft-Gault equation, based on the patient’s sex, age, serum creatinine, weight and height.

### TDM of ceftazidime/avibactam

Due to the continuous infusion administration of ceftazidime/avibactam, a random blood sample for its TDM was drawn during its infusion. Then, it was immediately placed on ice and transported to the laboratory for processing. The TDM of β-lactams is performed in our hospital and it is daily available from Monday to Friday. Upon arrival, sample was centrifuged, and both ceftazidime and avibactam concentrations were quantified using a validated high-performance liquid chromatography method with ultraviolet detection set at 290 nm, as previously described.^13,15^ The method demonstrated linearity within the range of 1.8–300 mg/L for ceftazidime and 2.5–50 mg/L for avibactam. Total plasma concentrations were measured, while unbound drug fractions are estimated based on previously reported protein binding values of 10% for ceftazidime and 7% for avibactam.^16^

## Results

### Clinical case (events summarized in Figures 1 and  2)

A 27-year-old Caucasian man from Siena (Tuscany, Italy) was diagnosed with AML on 10 May 102022. He had no significant comorbidities except for a smoking habit. His AML was characterized by WT1 overexpression, NPM1 type A mutation, FLT3-ITD mutation and a normal karyotype, placing him in the intermediate-risk category according to the 2022 European LeukemiaNet (ELN) guidelines.^17^ Initial presentation included anaemia, thrombocytopaenia and hyperleukocytosis (56 × 10^6^/L blasts).

**Figure 1.**
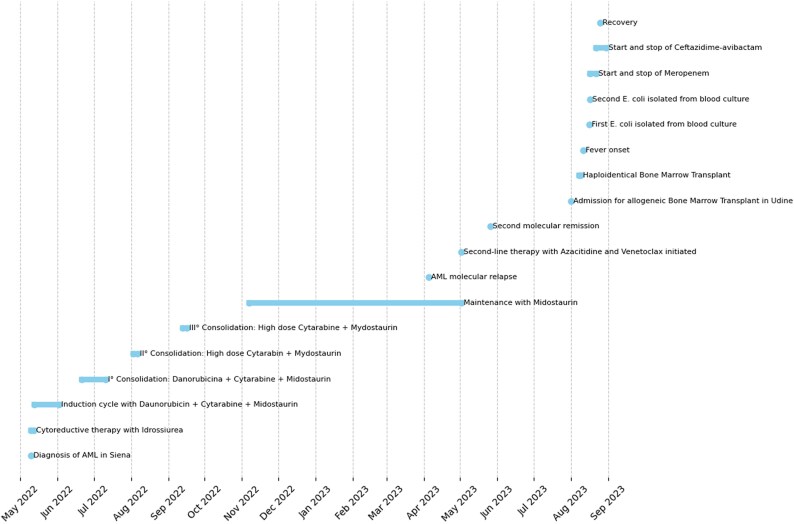
Clinical event timeline (all events).

**Figure 2.**
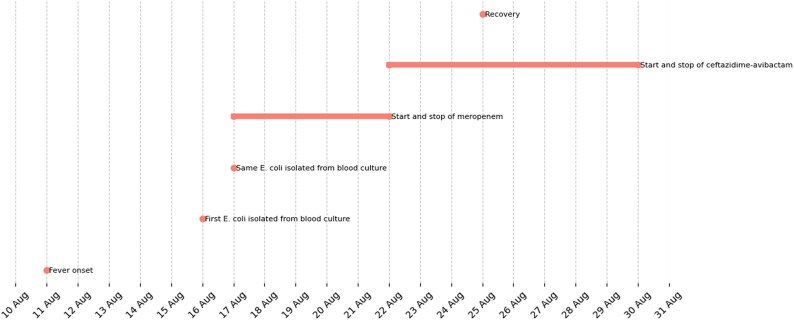
Event timeline from fever to recovery (negative blood culture).

Following induction chemotherapy with daunorubicin, cytarabine and midostaurin (May 13th to June 2nd), the patient achieved remission and completed three consolidation cycles (June 21st to September 16th treated at Udine University Hospital, Italy). However, a molecular relapse occurred on April 2023 after maintenance therapy initiation, prompting second-line treatment with azacitidine and venetoclax under the Italian GIMEMA AML2521 protocol (May 2023).^18^ A second complete remission was achieved before allogeneic HSCT on 8–9 August 2023, at Udine Hospital.

The transplantation, using a haploidentical donor (his 21-year-old brother), was performed following a myeloablative thiotepa-busulfan-fludarabine conditioning regimen. Notably, the patient was colonized with VIM-producing *Klebsiella pneumoniae*, raising concerns about the development of BSI (bloodstream infection) caused by a MDR Gram negative. Two days after undergoing allogeneic bone marrow transplantation (Allo-BMT), the patient developed a fever and was started on a continuous infusion of piperacillin/tazobactam (18 g/day). In the absence of any prior documented *E. coli* BSI, on 16 August 2023 a breakthrough bloodstream infection caused by *E. coli* (with a CZA MIC of ≤1 mg/L) was identified during ongoing piperacillin/tazobactam therapy (Table 1 for complete antibiogram). Blood cultures were also positive on 17 August 2023. An immediate decision was made to switch antibiotic therapy by starting meropenem; however, the patient was switched from meropenem to ceftazidime/avibactam (2.5 g every 8 h via continuous infusion) on Day 3, for 9 days, of treatment to implement a carbapenem-sparing regimen. First negative blood culture on 25 August 2023.

### Creatinine clearance and ARC values

In our patient, the creatinine clearance on the day CZA therapy was initiated was 236 mL/min (creatinine 0.53 mg/dL), with a maximum value of 255 mL/min (creatinine 0.49 mg/dL) observed on Day 7 of treatment and a minimum of 227 mL/min (creatinine 0.55 mg/dL) on Day 5.

### TDM results

TDM was performed on the third day of therapy with ceftazidime/avibactam: ceftazidime levels were 29.57 mg/L, while avibactam levels reached 5.52 mg/L.

### WGS and conjugation experiments

Molecular analysis, performed by WGS, showed the presence of genes conferring resistance to β-lactams (*bla_DHA-1_*), macrolides (*mphA*), fluoroquinolones (*qnrB4*) and trimethoprim (*dfrA7*). Plasmid-mediated fluoroquinolone resistance was confirmed by the presence of *qnrB4*. Additionally, plasmids IncFIB and IncFII were identified along with insertion sequences IS6100 (IS6 family), MITEEc1, IS100 (IS21 family), ISEc31 (IS3 family), IS421 (IS4 family) and IS26 (IS6 family). According to the MLST Achtman scheme, the strain belonged to ST442. *E. coli* serotyping, predicted by SeroType Finder, showed O174:H9 serotype, with the fimbria variant *fimH32* and *fumC95*. Several virulence-associated genes (*csgA*, *fdeC*, *fimH*, *fyuA*, *hlyE*, *irp2*, *lpfA*, *nlpI*, *terC*, *yehA*, *yehB*, *yehC*, *yehD*) were also detected. Conjugation experiments was performed to demonstrate the successful transfer of *bla_DHA-1_* from *E. coli* ST442 to *E. coli* J53 and, its really location on the plasmid.

## Discussion

This clinical case underscores the value of comprehensive genomic characterization, encompassing resistance genes, mobile genetic elements, plasmids and virulence factors, in guiding targeted therapy, particularly in complex or refractory infections; however, its routine application remains challenging in clinical practice due to the time-consuming nature of whole-genome sequencing.

The detection of multiple virulence-associated genes, including *csgA*, *fdeC*, *fimH*, *fyuA*, *hlyE*, *irp2*, *lpfA*, *nlpI*, *terC*, *yehA*, *yehB*, *yehC* and *yehD*, underscores the multifaceted pathogenic potential of the isolate under study. The csgA gene encodes the major subunit of curli fimbriae, which are extracellular amyloid fibres known to facilitate adhesion and biofilm formation, critical steps in bacterial colonization.^19^ Similarly, *fimH*, a mannose-specific adhesin located at the tip of type 1 fimbriae, plays a pivotal role in mediating attachment to host urothelium cells, promoting also formation of intracellular biofilm.^20^ The presence of *fyuA* and *irp2* are essential for iron acquisition, thereby enhancing bacterial survival and virulence, these are known to be involved in the synthesis of yersiniabactin components.^21^ Additionally, the *hlyE* gene encodes a novel pore-forming haemolysin that promotes host cell lysis and immune evasion, thereby enhancing bacterial survival and pathogenesis during intestinal colonization.^22^ Genes such as *yehA-D* and *nlpI* are implicated in adhesion and cell division respectively.^23,24^ Collectively, this constellation of virulence determinants illustrates a complex and coordinated strategy employed by the pathogen to adhere, invade and sustain infection within the host.

Indeed, Gram-negative bacteria with inducible AmpCs are particularly challenging because, more often, *in vitro* susceptibility does not correlate with clinical efficacy of treatment.^25^

This pattern necessitates careful antimicrobial selection in high-risk patients, particularly post-transplant patients. TDM played a pivotal role in optimizing the antibiotic regimen, particularly in the context of the patient’s ARC. Studies have shown that ARC significantly affects drug clearance in critically ill patients, including those with haematological malignancies, as seen in this case, and this may result in suboptimal drug levels,^26^ also influencing specifically ceftazidime/avibactam concentrations.^13,27^ In high-risk populations, such as neutropenic patients with ARC, pharmacokinetic/pharmacodynamic (PK/PD) optimization is crucial. Continuous infusion of β-lactams, like ceftazidime/avibactam, has been shown to improve PK/PD target attainment in critically ill patients.^28^ The TDM results on the third day of therapy showed ceftazidime plasma levels of 29.57 mg/L, which are markedly higher than the MIC of ≤1 mg/L for *E. coli*,^29^ corresponding to a fCss/MIC ratio > 25 and suggesting sustained bactericidal exposure. Concurrently, avibactam concentrations reached 5.52 mg/L, yielding a C/MIC ratio > 5, thereby exceeding the pharmacodynamic threshold considered effective for β-lactamase inhibition. These findings are in line with recent evidence showing that optimal joint PK/PD target attainment, defined as fCss/MIC ≥ 4 for ceftazidime and fCss > 4 mg/L for avibactam, is associated with significantly improved microbiological eradication and reduced risk of failure in patients treated with continuous infusion ceftazidime–avibactam.^30^ This result is consistent with recent data that demonstrate how continuous infusion strategies improve PK/PD target attainment, particularly in patients with ARC.^31^

With regard to these aspects, avoiding carbapenem overuse was a priority in this patient to reduce the risk of microbiota disruption and prevent plasmid-mediated AmpC β-lactamase induction, which could exacerbate resistance development. Carbapenem-sparing strategies, such as ceftazidime/avibactam, were considered crucial not only for preserving the gut microbiome (ceftazidime does not have anti-anaerobic activity) but also for minimizing the induction of resistance genes.^32^ While the patient was colonized with VIM-producing *K. pneumoniae*, no BSI due to this organism occurred during the clinical course. This outcome is noteworthy, considering that rectal colonization with CPE is a well-established risk factor for subsequent invasive infections in patients.^33^

Several factors may have contributed to preventing *K. pneumoniae* VIM BSI in this case. First, the early switch from meropenem to ceftazidime/avibactam may have reduced selective pressure favouring CPE overgrowth. Second, ceftazidime/avibactam’s lack of anti-anaerobic activity may have helped preserve gut microbiota diversity, which is known to protect against multidrug-resistant organisms.^34^ Although we cannot definitively demonstrate a causal relationship, we believe that prompt antimicrobial adaptation and microbiome-sparing strategies contributed to the containment of colonization without progression to invasive disease. Although no microbiome analysis was performed in this case, the use of ceftazidime/avibactam, which lacks significant anaerobic activity, was chosen with the aim of minimizing potential microbiota disruption compared to carbapenems, a factor that has been implicated in the pathogenesis of aGVHD following allogeneic stem cell transplantation especially in the first two weeks after BMT.^12^

Moreover, several similar cases have been reported in the literature, both in single-centre and multi-centre observational studies, indicating that ceftazidime/avibactam may represent an effective treatment option for infections, including those caused by KPC-producing organisms and carbapenem-resistant *P. aeruginosa*, in patients with haematological malignancies, particularly when initiated early before clinical deterioration.^35,36^

As reported above, a significant increase in carbapenem-resistant isolates has been observed. These infections have a considerable clinical impact, as the 30-day mortality rate in patients with BSIs caused by CPE can be extremely high compared to patients with Enterobacterales bacteraemia without resistance mechanisms or even those producing ESBL.^37^ Ceftazidime–avibactam is an antibiotic combination active against various species of Enterobacterales, including carbapenem-resistant strains. The switch from meropenem to ceftazidime/avibactam was justified by the susceptibility profile of the isolated *E. coli* strain, which was resistant to piperacillin/tazobactam but susceptible to ceftazidime/avibactam. This approach helped avoid the induction of AmpC and other plasmid-mediated resistances, reducing the risk of horizontal resistance transfer among Enterobacterales. Although carbapenems are effective against AmpC producers, using ceftazidime/avibactam provides a viable alternative with a more favourable resistance profile and opportunities for individualized dosing via TDM. In this case, TDM of meropenem was not performed due to logistical constraints; however, we acknowledge that it could have supported optimized, individualized dosing and may have been beneficial. Moreover, while ceftolozane/tazobactam (C/T) might be effective against some AmpC-producing *E. coli* strains, the MIC in this case supported the use of ceftazidime/avibactam as the more potent option.^32^ The use of continuous infusion of ceftazidime/avibactam may provide an additional benefit by potentially maintaining more stable drug concentrations and reducing the risk of underdosing in patients with ARC; however, since TDM was performed only on the third day, this assumption cannot be confirmed with certainty. For instance, ceftazidime levels may remain higher than expected, while avibactam may be cleared more rapidly due to renal transport mechanisms, potentially altering the intended 4:1 drug ratio.^38^

In conclusion, this case highlights the importance of carbapenem-sparing strategies, particularly in patients undergoing allogeneic stem cell transplantation, proved effective in successfully treating a multidrug-resistant *E. coli* infection probably without compromising the patient’s microbiome or increasing the risk of complications such as GVHD. Optimizing PK/PD parameters through TDM, especially in neutropenic patients with ARC, is essential for achieving therapeutic efficacy with agents like ceftazidime/avibactam. Individualized dosing regimens based on real-time pharmacokinetic data may provide a safer and more effective approach to managing infections caused by multidrug-resistant organisms, while minimizing the risk of resistance development associated with carbapenem overuse.
